# A transcriptome-SNP-derived linkage map of *Apios americana* (potato bean) provides insights about genome re-organization and synteny conservation in the phaseoloid legumes

**DOI:** 10.1007/s00122-017-3004-3

**Published:** 2017-10-25

**Authors:** Jugpreet Singh, Scott R. Kalberer, Vikas Belamkar, Teshale Assefa, Matthew N. Nelson, Andrew D. Farmer, William J. Blackmon, Steven B. Cannon

**Affiliations:** 1 ORISE Fellow, Corn Insects and Crop Genetics Research Unit, USDA-ARS, Ames, IA 50011 USA; 2 Crop Genome Informatics Laboratory, Corn Insects and Crop Genetics Research Unit, USDA-ARS, Ames, IA 50011 USA; 3 Department of Agronomy and Horticulture, University of Nebraska-Lincoln, Lincoln, NE 68583 USA; 4 Royal Botanic Gardens, Kew, Wakehurst Place, Ardingly, West Sussex RH17 6TN UK; 5 National Center for Genome Resources, Santa Fe, NM 87505 USA; 6 5097 Studley Rd, Mechanicsville, VA 23116 USA

## Abstract

**Key message:**

**We report a linkage map for** ***Apios americana*** **and describe synteny with selected warm-season legumes. A translocation event in common bean and soybean is confirmed against** ***Apios*** **and** ***Vigna*** **species.**

**Abstract:**

Apios (*Apios americana;* “apios”), a tuberous perennial legume in the Phaseoleae tribe, was widely used as a food by Native Americans. Work in the last 40 years has led to several improved breeding lines. Aspects of the pollination biology (complex floral structure and tripping mechanism) have made controlled crosses difficult, and the previous reports indicated that the plant is likely primarily an outcrosser. We used a pseudo-testcross strategy to construct a genetic map specific to the maternal parent. The map was built using single-nucleotide polymorphism markers identified by comparing the expressed sequences of individuals in the mapping population against a de novo maternal reference transcriptome assembly. The apios map consists of 11 linkage groups and 1121 recombinationally distinct loci, covering ~ 938.6 cM. By sequencing the transcriptomes of all potential pollen parents, we were able to identify the probable pollen donors and to discover new aspects of the pollination biology in apios. No selfing was observed, but multiple pollen parents were seen within individual pods. Comparisons with genome sequences in other species in the Phaseoleae showed extended synteny for most apios linkage groups. This synteny supports the robustness of the map, and also sheds light on the history of the Phaseoleae, as apios is relatively early diverging in this tribe. We detected a translocation event that separates apios and two *Vigna* species from *Phaseolus vulgaris* and *Glycine max*. This apios mapping work provides a general protocol for sequencing-based construction of high-density linkage maps in outcrossing species with heterogeneous pollen parents.

**Electronic supplementary material:**

The online version of this article (doi:10.1007/s00122-017-3004-3) contains supplementary material, which is available to authorized users.

## Introduction

Molecular markers and genetic maps have been crucial in genetic diversity analysis and in speeding up the breeding efforts in many crops (Staub et al. 1996; Xu and Crouch 2008; Dhillon et al. 2009; Roy et al. 2011; Jiang 2013; Belamkar et al. 2016). The recombination-based ordering of molecular markers can help in dissecting genotype–phenotype relationships and in map-based cloning. Maps are also important for genome sequence assembly (for scaffold positioning along the genomes) and for evolutionary analysis through synteny comparisons between related species (Kujur et al. 2015; Collard et al. 2005; Fierst 2015; McConnell et al. 2010; Muchero et al. 2009; Bertioli et al. 2009). Development of molecular markers is an important initial step towards map-based analysis of any species. A wide range of DNA-based marker techniques have been developed in the last 3 decades; these often required significant time commitments and resources to develop sufficient numbers of markers for meaningful genetic studies. Recently, sequencing-based methods that combine discovery and genotyping in a single step are superseding conventional marker development techniques and have substantially reduced laboratory investments. These methods can facilitate genome-wide identification of polymorphic markers in a cost-effective and timely fashion with or without the availability of previous genetic knowledge about a particular species. Whole-genome sequencing (Hillier et al. 2008), exome capture (Ng et al. 2009), RNA sequencing (Hansey et al. 2012), and sequencing DNA fragments after digestion with various restriction enzymes (reviewed by Davey et al. 2011) can produce thousands of polymorphic markers for genotyping and linkage analysis. These techniques follow diverse protocols for variant detection and can be easily adopted in less-characterized species.

Apios (*Apios americana*), also known as “potato bean” or “American groundnut”, is a nitrogen fixing legume, with a native range spanning most of eastern North America. Both diploid (2*n* = 2*x* = 22) and triploid (3*n* = 3*x* = 33) forms exist for apios (Seabrook and Dionne 1976). Apios has a haploid genome size of ~ 830 Mbp (Bai et al. 2012; Belamkar et al. 2016). Triploid populations are evidently sterile (Bruneau and Anderson 1988) and propagate through vegetative means. Diploid populations also have been reported to show at least partial self-incompatibility, resulting in low rates of self-pollination. Bruneau and Anderson (1988) suggest that Apios shows partial gametophytic self-incompatibility (GSI), based both on the low (but nonzero) rate of self-pollination that they observed, and on floral features associated with GSI, including small papillate stigmas, stigmatic exudate, stylar inhibition of pollen tube growth, and binucleate pollen. A floral tripping mechanism, in which the pistil is dislodged from the enclosing keel petal and then recoils to place the stigma back against the pistil’s shaft, is triggered either by pollinators or through manual interference (Bruneau and Anderson 1988). This tripping is necessary for successful cross-pollination and hence sexual propagation between apios plants. Although selfing has been observed in apios, the rate of selfing was low in controlled crosses reported by Bruneau and Anderson (1988), who conclude that it is “likely that *A. americana* is partially self-compatible or that it possesses a pseudoself compatibility system.”

Apios is in the Phaseoleae tribe, among many other economically important legume species, including soybean (*Glycine max*), common bean (*Phaseolus vulgaris*), and cowpea (*Vigna unguiculata*) (Lavin et al. 2005; Stefanovic et al. 2009). However, the capacity to produce edible underground storage tubers (Belamkar et al. 2015) distinguishes it from most well-studied food legumes. Apios tubers are rich in both starch and protein, and the plant is also of interest because of its perenniality and its tolerance of flooding and of acidic soils (Beardsley 1939; Blackmon and Reynolds 1986; Belamkar et al. 2015; Yangcheng et al. 2016). Apios has a well-documented history of service as a food crop in North America (Beardsley 1939), and more recently have been grown commercially in northeast Asia (Kikuta et al. 2011). The preservation of synteny between apios and well-characterized legume crops like soybean and common bean may help researchers to accelerate the genetic improvement in apios and its development as a crop. Conserved synteny patterns have been discovered among different legumes using whole-genome sequences or linkage-based linear arrangement of suitable molecular markers (Cannon et al. 2006; McConnell et al. 2010). Synteny plots based on mapped, sequence-based markers provide a suitable initial method for synteny characterization (Muchero et al. 2009; Bertioli et al. 2009).

This study presents the development of a high-density single-nucleotide polymorphism (SNP) marker-based linkage map for apios. A de novo reference transcriptome was generated using RNA-Seq data from the maternal parent (AA-2155) to align expressed sequences of individuals of the F_1_ mapping population (*n* = 73) for SNP detection and genotyping. A one-way pseudo-testcross strategy was implemented to map SNP segregation patterns. We further assessed the genome architecture and evolutionary patterns in apios through comparative sequence analysis of linearly ordered markers against other phaseoloid legumes.

## Materials and methods

### Plant material

Dr. William J. Blackmon provided the F_1_ seed material for this study. The seeds were obtained from an open-pollination breeding collection, with a known maternal parent, AA-2155, and a set of 37 genotypes genetically characterized through transcriptome sequencing (Belamkar et al. 2016) that could serve as potential pollen sources. No other apios plants were growing in the vicinity of the maternal parent. The maternal parent, AA-2155, is a high yielding accession and produces a large number of medium-sized child tubers (tubers produced in the current growing year) and a single large-sized mother tuber (the tuber from which shoots and stolons originate in the current growing year). The F_1_ seeds were planted in germination trays and kept in the greenhouse to facilitate germination and growth under controlled conditions. The surviving healthy seedlings, with first trifoliate leaves completely open, were transplanted in the field at the North Central Regional Plant Introduction Station (NCRPIS) in Ames, IA in 2014. In the field, individual plants were grown in rows, with 2-m separation between rows and half-meter separation between plants in the row. Each plant was provided a bamboo stake for support. Young leaf tissue was harvested as described below. After frost, tubers were dug, measured, and stored at 4 °C through the winter for subsequent grow-out and evaluation—though the mapping results depend only on tissue collected during the first season.

### Cytology and ploidy assessment

Because both diploid and triploid apios accessions have been reported, we checked ploidy of the maternal parent with microscopic visualization of root cell preps. Root tips for cytological preparations were collected from germinated seedlings of apios and fixed in 1:3 acetic acid: ethyl alcohol for 24 h. Fixed root tips were hydrolyzed in 2 ml of 1 N HCl for 15 min in a water bath at 60 °C and stained with Schiff’s reagent for 45 min (Sharma and Sharma 1980). A drop of 45% acetic acid was added to the root tips, and a cover slip was placed over the root tip, and gently pressed to disperse the tissue and cells. The cover-slipped slide was placed on CO_2_ freezing unit to rapidly freeze the slipcovered area with stream of liquid CO_2_. The coverslip was removed using a razor blade and the slide was plunged into 95 and 100% ethanol for 1 min each. The slide was transferred to pure xylene for 1 min and a drop of Permount was added and cover-slipped again. The slides were viewed using a Zeiss Axioplan II compound upright microscope (http://www.carlzeiss.com). Cells with well-scattered chromosomes from root tips were observed and chromosome numbers were counted.

### RNA extraction and sequencing

Young leaves were harvested from the parental and F_1_ lines and immediately frozen in liquid nitrogen. The frozen tissue was stored at − 80 °C until RNA extraction was performed. Frozen leaf tissues were homogenized into fine powder for each separate individual. Total RNA was extracted with Qiagen RNeasy^®^ Plant mini kit per the manufacturer’s instructions, and was purified with Qiagen RNA purification kit. RNA quality was assessed using a NanoDrop spectrophotometer and Agilent 2100 Bioanalyzer. High-quality RNA was used for library preparation and sequencing. Sequencing libraries were prepared using TruSeq RNA Sample Preparation kits (Illumina Inc.) as described by the manufacturer. The libraries were sequenced on the Illumina Hi-seq platform to obtain 50-bp single-end reads for each individual. In addition, RNA from the maternal parent was used to prepare a separate RNA-Seq library to generate 100 bp paired-end sequencing reads from a single lane of the Illumina Hi-seq platform. The sequencing reads of the maternal parent were used to build a de novo reference transcriptome assembly. The library preparation and sequencing were performed at the DNA facility, Iowa State University, Ames, IA.

### Read processing and de novo transcriptome assembly

Sequencing analysis involved various bioinformatics software for quality control, reference generation, and identification of polymorphic markers. The single-end and paired-end reads were separately processed for quality using fastqc (http://www.bioinformatics.babraham.ac.uk/projects/fastqc/) and Trimmomatic (Bolger et al. 2014). Raw sequencing reads were quality filtered by removing adapter/primer sequences followed by trimming low-quality bases from 3′ to 5′ ends (quality score > 20). Different minimum read length criteria were provided for single-end and paired-end reads (20 for 50 bp single-end reads and 35 for 100 bp paired-end reads). The resulting reads were further processed to eliminate low-quality reads (quality score < 20). Barcode information was used to separate the sequenced reads for each genotype and reads were trimmed to remove the corresponding barcode sequences.

The high-quality paired-end reads from the maternal parent were used to perform de novo transcriptome assembly with Trinity v 2.0.2 (Haas et al. 2013), using default settings. The assembled transcripts were examined for quality by comparing against peptide sequences from *P. vulgaris* (v 1.0) and *G. max* (Glyma.Wm82.a2) using blastx implemented in the BLAST+ (Camacho et al. 2009) package with e-value 1e−10. For functional annotation, the resulting transcript sequences were used to extract the open reading frames (ORFs) using Transdecoder v 2.0.1 (https://transdecoder.github.io/) with parameters, “–search_pfam” and “-m 67″. The transcripts were scanned against the UniProt database with parameters (-e-value 1e−5 -num_threads 6—-outfmt 6) (ftp://ftp.uniprot.org/pub/databases/uniprot/current_release/knowledgebase/complete/uniprot_sprot.fasta.gz) and searched for protein domains by comparing against the Pfam database. Perl and Unix shell scripts were used to extract the best matches in terms of sequence identity and coverage.

The de novo transcriptome assembly generated from maternal parent AA-2155 contained a large number of redundant contigs due to the presence of splice variants and likely assembly errors (genes represented by smaller incomplete contigs or paralogs), which could complicate the downstream analysis. The longest spliced variant was retained from each gene model to develop a set of unique transcripts. The remaining contigs were compared against each other to identify redundant contigs (sequence similarity ≥ 95%). Only the longest contigs from the latter analysis were used and placed with other unique contigs. The remaining contigs were compared again using the CD-Hit-EST program (Li and Godzik 2006) and the resulting unique sequences were used to create a “refined” reference transcriptome, which we used for aligning the mapping populations and for SNP calling. The refined transcriptome assembly is available at the Legume Information System Data Store, https://legumeinfo.org/data/public/Apios_americana (Dash et al. 2015).

The transcript sequences in the refined assembly were provisionally ordered relative to *P. vulgaris* chromosome references to build single scaffolds representing the candidate apios chromosomes. The remaining unmatched transcripts were concatenated to build a separate pseudo-scaffold. A total of 12 apios scaffolds (11 *P. vulgaris* chromosome specific and 1 unmatched transcript sequences) were used as a pseudo-reference for variant calling.

### SNP calling

High-quality reads from the mapping population were aligned against the pseudo-reference transcriptome generated in the previous steps to develop an SNP database. We first tested the precision of mapping and SNP calling from “unreduced” (original de novo assembly) and “refined” (filtering redundant sequences) reference transcriptome sets by comparing alignment statistics with a set of random genotypes whose leaf transcriptomes were sequenced in our previous study (Belamkar et al. 2016). These genotypes were grown under controlled conditions in a greenhouse, and tissue harvesting and RNA extraction were performed similar to that defined for the current publication. Based on this analysis, the reduced transcriptome was used as a pseudo-reference for SNP calling and genotyping. The Alpheus pipeline (Miller et al. 2008) was used for mapping and variant identification for each line in the mapping population. The positional information for each nucleotide in the reference transcriptome and the alternate allele were recorded in the variant call format (VCF). The bioinformatics tool “vcftools” (Danecek et al. 2011) was used to determine the high-quality SNP locations using the following criteria: minimum read depth ≥ 5, variant allele frequency ≥ 20%, and base quality (read depth) for variant allele ≥ 10. The positions showing no polymorphism between the reference and the alternate alleles and indels were excluded from further analysis. Some positions were called as polymorphic due to the presence of alternate alleles in less than 10% of the mapping population lines (minor allele frequency ≤ 0.1). These SNP calls were considered as probable sequencing errors and were removed from the data set. The remaining SNP markers were further filtered using missing allele percentage ≤ 10%. Each SNP marker was assigned a unique ID based on the original pseudo-chromosome name and its corresponding position on the pseudo-chromosome sequence. The SNP data set is available through the Legume Information System Data Store, https://legumeinfo.org/data/public/Apios_americana (Dash et al. 2015).

### Parentage analysis

The F_1_ progeny in this study were generated from an open-pollination breeding design with known maternal parent and unknown pollen sources. To identify the candidate pollen parents, we obtained the transcriptome sequences from the 52 genotypes used in the breeding program (Belamkar et al. 2016). These transcriptome sequences were aligned to the reference assembly as described previously. The positions of resulting SNP calls were matched with the positions of high-quality SNPs identified in the F_1_ population. Subsequently, we generated a merged SNP data set that consisted of common SNPs in the mapping population and genotypes in the breeding population. Of the 52 candidate pollen parents, only 37 were present in the field at the time of pollination, so the remaining 15 that were not present served as negative controls in the parentage analysis.

The data set generated above was used to estimate the log-likelihood scores for parentage assignments in the Cervus v 3.0.7 software (Kalinowski et al. 2007). Cervus used a three-step approach for parent assignments that involves an initial calculation of allele frequencies, expected heterozygosity, and polymorphic information content (PIC) for each SNP marker. The parental assignment is simulated in the next step to calculate the threshold log-likelihood values. The simulations were performed for 10,000 offspring using a known mother and 52 candidate fathers (including 15 negative controls), with default 1% genotype error rate and two confidence levels of 95% (strict) and 85% (relaxed). The genotypes of the loci were derived from the allele frequency analysis in the first step. The information from above two steps was used to perform the parentage assignment with known mother and 52 candidate fathers. The parentage assignment results were compared with the field map as an accuracy check, under the assumption that closely planted genotypes would be likely pollen sources for cross-pollination.

### Linkage analysis

Meiotic events in an F_1_ population resulting from cross-pollination between two heterozygous parents can provide four different types of segregation patterns for bi-allelic markers as follows: (A) homozygous maternal mated with heterozygous paternal (‘*nn x np*’), (B) heterozygous maternal crossed to homozygous paternal (‘*lm x ll*’), and (C) both parents are heterozygous at a particular location (‘*hk x hk*’). The first two crossing types will provide a 1:1 segregation ratio for homozygous and heterozygous genotypes. However, the markers in (C) will display 1:2:1 segregation. The SNP markers were converted into a mapping software usable format based on the segregating marker types for further linkage calculations and map construction. A pseudo-testcross mapping scheme was used to calculate the pair-wise recombination and linkage distances between the high-quality SNP markers exhibiting the maternal specific (‘*lm x ll*’ *and* ‘*hk x hk*’) segregation patterns. Marker loci were grouped separately for each segregation type (maternal backcross and F_2_ heterozygous) using the MSTmap program (Wu et al. 2008). Markers belonging to the same haplotype groups were identified and only a single marker with the least missing information was chosen from any particular haplotype group to represent the recombination event. Markers were initially grouped using a high threshold (*P* < 1e−9) value to identify stable grouping pattern. The process was repeated several times using stricter threshold values to define high confidence groups for apios map construction. The markers within each group were used for linkage phase determination, ordering and recombination analysis using Joinmap v 4.1 (Van Ooijen 2006) with a cross-pollinated (CP) design and “regression mapping” algorithm. We also tried the “maximum likelihood” algorithm for calculation of genetic distances among markers, but it produced large linkage groups by assigning a probable position for every marker in the data set. Thus, we preferred the “regression mapping” to calculate the recombination frequencies and genetic distances using the Kosambi mapping function. All linkage groups in the final map were generated with an LOD of 4 and recombination frequencies smaller than 0.35. The linkage map graphics were generated using Mapchart v 2.2 (Voorrips 2002).

Given the unconventional nature of the mapping population, we independently developed a second linkage map using the MultiPoint v3.3 software (MultiQTL Ltd, Haifa, Israel). This software implements the ‘evolutionary optimization strategy’ (Mester et al. 2003) to conduct multi-locus ordering of linkage groups. Our approach was similar to that reported by Kroc et al. (2014) with some modifications, as follows. The F_2_ population option was used and the initial quality control resulted in the removal of one mapping population individual (AA-44) due to a high level of missing data points (34%). No further filtering of marker segregation ratios beyond that already carried out during JoinMap analyses was implemented. Redundant markers were first removed before clustering at recombination frequency threshold, rf = 0.15, which yielded a set of initial linkage groups. These linkage groups were then subjected to jack-knife resampling to identify markers that had a destabilizing effect on locus order, which were then removed. Only the remaining high-quality ‘skeleton’ markers were used to calculate locus order. Affinities between the initial linkage groups were identified that allowed smaller linkage groups to be appended to larger linkage groups to form the final linkage groups.

### Comparative analysis

The syntenic conservation of apios with *P. vulgaris*, *G. max*, *Vigna radiata*, and *Vigna angularis* was evaluated by identifying the best sequence match of mapped SNP sequences with available genome sequences from the above four species. First, a 200-bp sequence associated with each mapped locus was retrieved from the apios pseudo-chromosomes with a Python script. The sequences were kept in the same order as markers on each linkage group. Next, a scaffold of ‘N’s that proportionally reflected the genetic distance (cM) on each linkage group in terms of physical distance (e.g., a linkage group of 119.5 cM will represent the 119,500 bp long scaffold) was created. Finally, the ‘N’s at each SNP marker position (cM map unit) in the scaffold were replaced with corresponding 200-bp marker sequences using a Perl script (e.g., marker position 39.5 cM will lead to replacement of ‘N’s at position 39,500 + 200 with the SNP sequence). These scaffolds represent the linear order of sequences from mapped SNP markers separated by ‘N’s with assigned coordinates for each base that depend on the genetic distances between markers and the total size of a linkage group. Separate scaffolds were generated for each linkage group (11 scaffolds) and combined as a single multifasta file for genome-wide comparison.

The genome sequences for *P. vulgaris* (version 1.0; Schmutz et al. 2014) and *G. max* (Wm82.v2; Schmutz et al. 2010) were downloaded from Phytozome (Goodstein et al. 2011), while *V. radiata* (Kang et al. 2014) and *V. angularis* (Kang et al. 2015) genome sequences were retrieved from http://plantgenomics.snu.ac.kr/mediawiki-1.21.3/index.php/Main_Page. Only chromosome sequences were retained for each genome and any additional scaffolds were discarded for synteny analysis. The blastn program was used to compare the apios scaffold sequences against the chromosome sequences from these four warm-season legume species. The homology matches with e-value < 1e−10 and a minimum sequence overlap of 80 bp was used for synteny comparisons. The genomic coordinates for the best-matched sequences in query and subject sequences were used to generate dot-plot views for each genome comparison using “mummerplot” tool in the MUMmer software package (Kurtz et al. 2004).

## Results

### Reference generation and validation

**Fig. 1:**
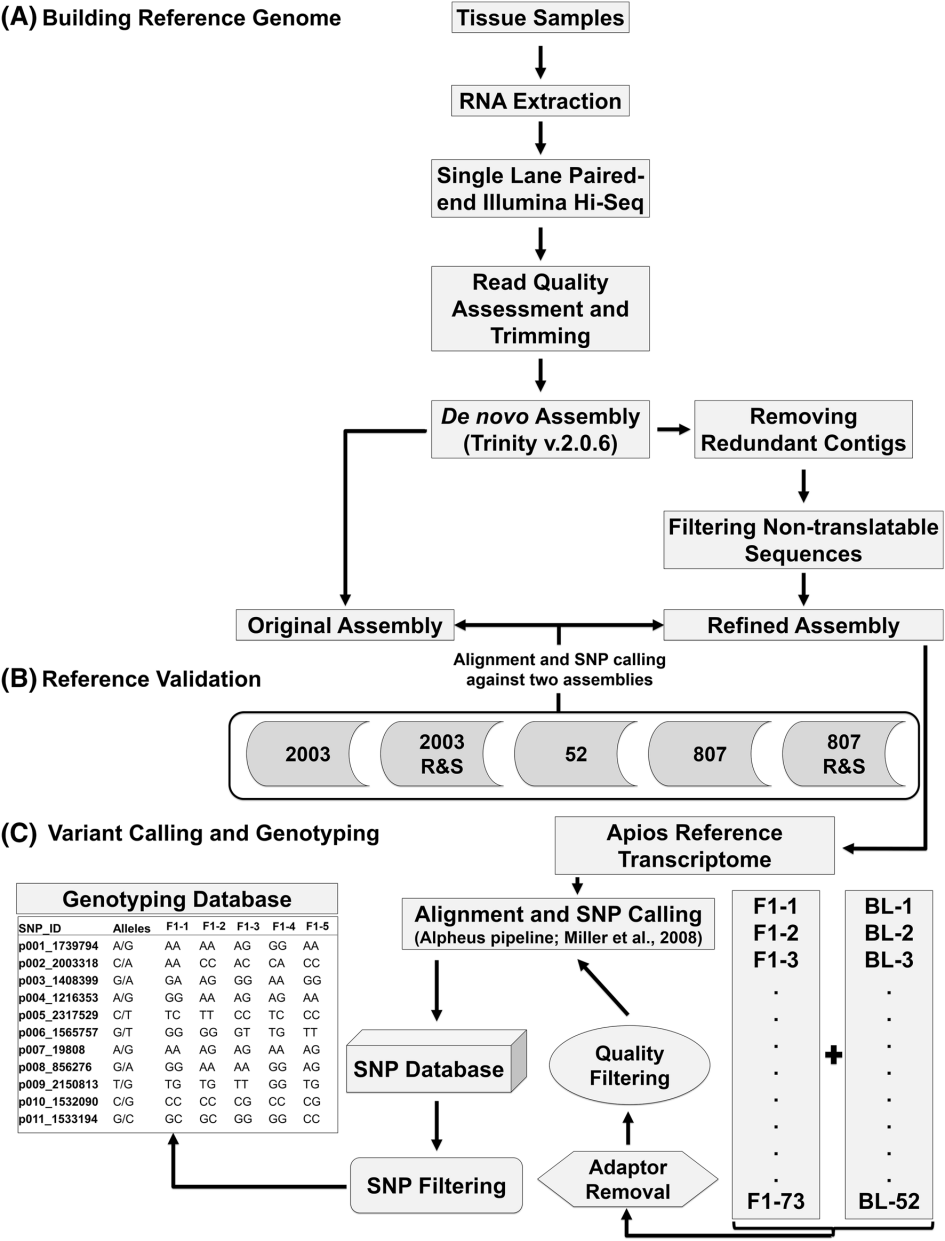
Flow chart describing various steps for reference transcriptome generation (A), validation (B), and single-nucleotide polymorphism (SNP) identification (C) in the F1 progeny and the parental breeding lines (BLs)

**Fig. 2:**
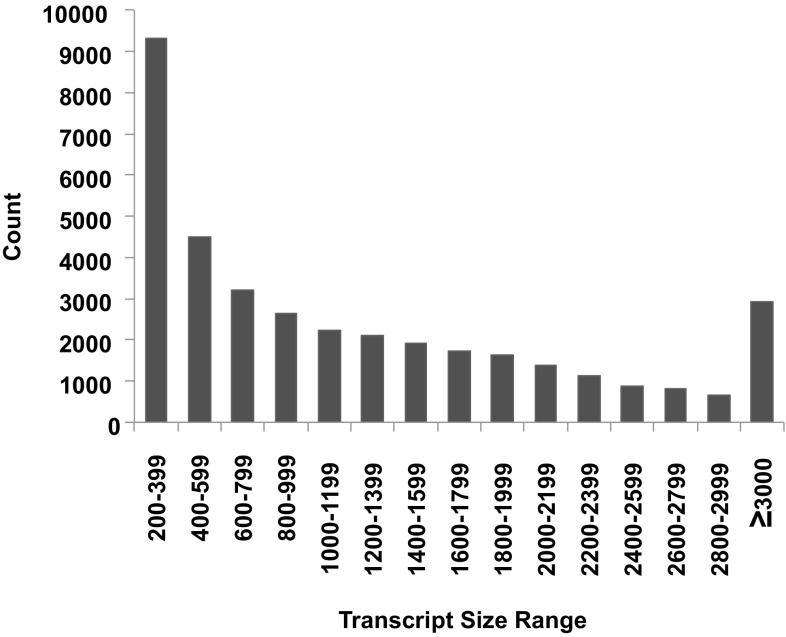
Frequency distribution of varying sized transcripts in the apios transcriptome assembly

The method for the development of a transcriptome-based reference sequence, mapping population alignment, and SNP genotyping involved multiple sequence processing steps (Fig. 1). A single-lane, deep RNA sequencing of pooled tissues from the maternal genotype (AA-2155) produced about 195 million paired-end reads, which reduced to approximately 189 million (2.8% reduction) after trimming read ends, filtering low-quality reads, and removing primer/adapter sequences. In addition, 2.7% of the reads were left without the matching pair after quality filtering and were treated as single-end for further analysis. A de novo transcriptome assembly was generated with -quality reads using the Trinity v 2.0.2 (Haas et al. 2013) assembler. The resulting apios transcriptome contained 157,733 contigs corresponding to 114,345 “gene” components (Table 1). The transcript lengths ranged from 201 to 20,244 bp having an N50 of 1558 bp and an average contig length of 892 bp. The assembly contained a large number of small-sized transcripts and about 40.5% of the contigs had a size range between 200 and 399 bp (Fig. 2). The number of transcripts decreased as the transcript size class increased. To avoid redundancy, the longest isoform sequence was retained for each gene model and all other splice variants were filtered out. A further identity check was performed at a threshold identity level of 95% to cluster the matching sequences. A comparison of apios transcriptome assembly against the full-length coding sequences of related warm-season legumes *P. vulgaris*, *G. max*, *V. radiata,* and *V. angularis* revealed about 52–79% of corresponding matches (Table S1). Similarly, the prediction of putative coding sequences in apios resulted in 56,488 open reading frames (ORFs), with 36,868 (65.2%) of the transcriptome having complete coding sequence coverage against the related legume sequences. The observations above support the relative completeness and high quality of the apios transcriptome.

**Table 1:** Summary of read statistics and transcriptome assembly

Summary	Count
Read statistics	
Total reads pair 1	195,129,626
Total reads pair 2	193,974,556
Cleaned read pairs	189,677,147
Cleaned reads unpaired	5,270,289
De novo assembly statistics	
Total number of assembled bases	140,779,251
Number of transcripts	157,733
Number of “gene” components	114,345
Size of longest transcript (bp)	20,244
Size of smallest transcript (bp)	201
Average transcript size (bp)	892
N50 ()	1558
Number of predicted peptides	56,488
Number of predicted peptides (refined assembly)	37,105
Mapping population statistics	
Number of F1 genotypes	73
Total read count	2,015,696,705
Average read count	27,612,284
Minimum read count	10,909,581
Maximum read count	53,507,402
Total mapped read count	1,529,865,674
Average mapped read count	20,957,064
Total uniquely aligned reads	1,525,660,847
Average uniquely aligned read	20,899,463
Total number of high-quality SNP	33,929

To validate the use of unreduced (without redundancy filtering) or refined (unique single-copy sequences) transcriptome assembly as the reference for mapping and SNP identification, we aligned RNA-Seq reads from pooled tissue samples of a set of genotypes used in our previous study (Belamkar et al. 2015). The alignment statistics indicated a larger proportion of reads uniquely aligned against the refined assembly than were aligned to the original assembly (Table S2). An additional variant call analysis was performed to compare the number of SNP calls generated from alignment against each transcriptome assembly. The total number of SNPs was greater when the original transcriptome assembly was used as a reference. However, the number of high-quality SNPs retained after stringent quality filtration was higher when using the refined assembly (212,769) than the original transcriptome assembly (136,098) (Table S3). This analysis indicated that removal of redundant sequences provided more precise alignments with an increased ability to capture a higher number of unique locations in the reference for potential SNP calling. The presence of redundant sequences in the reference increased the chances of low-quality variant calls, which are mostly unsuitable for further genetic analysis. Thus, we used the refined transcriptome assembly to build a pseudo-reference for alignment and SNP calling in the F_1_ mapping population.

### Cytology and ploidy assessment

Although we anticipated that the crosses in this study were among diploid plants, we checked the chromosome spreads to confirm the ploidy and to assess possible chromosomal abnormalities that might be reflected in a genetic map—such as unusually small, large, or acrocentric chromosomes. At the level of resolution of our microscopy, we observed no obvious abnormalities (Supplementary Fig. 1). We count 22 meiotic chromosomes, with roughly similar sizes.

### SNP genotyping

The leaf transcriptomes of 73 F_1_ lines were sequenced to generate approximately 2 billion reads, with an average yield of approximately 27 million reads per genotype (Table 1). The alignment percentage ranged between 60 and 82%, with a 76% average alignment rate. Mostly, the mapped reads have unique alignments with a small ambiguous mapping rate. Variants were scored using the alignment information to identify the SNP markers in the population. These SNP markers were filtered using different parameters to retain only high-quality variant types. The SNPs were finally filtered for their representation in at least 90% of the mapping population lines. A final count of 33,929 high-quality SNPs remained after stringent filtering. The SNPs show a higher number of transition (61.7%) than transversion events (38.3%). The most frequent allele variant was the transition of C ↔ T (17.5% of C to T, and 13.8% of T to C) followed by the transition of A ↔ G (13.9% of A to G, and 16.4% of G to A). Among the transversions, A ↔ T events had the highest representation (6.1% of A to T, 6.0% of T to A) followed by the G to T (5.0%) and C to A (4.9%) events. The C ↔ G transversion events were fewest (3.7% of C to G, 3.8% of G to C) in the SNP data set. The frequencies of the T (26.3%) and A (25.9%) alleles were comparatively higher than those for the C and G alleles (24.0 and 23.6%, respectively). The ratio of C and G alleles in a homozygous state was higher than for the heterozygous state (the homozygous/heterozygous ratio was 1.01 and 1.05 for C and G, respectively), but an opposite trend was observed for A and T alleles (the homozygous/heterozygous ratio was 0.92 and 0.96 for A and T, respectively).

An analysis of allelic segregation types in the data set revealed that markers with maternal segregation types (‘*lm x ll*’) were most common (51.1%), followed by the ratio types resulting from a cross between heterozygous loci (‘*hk x hk*’) in the two parental genotypes (35.6%). The remaining 13.3% of markers showed a male genotype-specific segregation pattern (‘*nn x np*’) obtained from crosses between homozygous maternal and heterozygous paternal genotypes.

### Parentage analysis

For identification of a putative pollen source for each F_1_ individual, a new SNP data set was created to perform the parentage analysis. The results of parentage analysis suggest multiple pollen sources as progenitors for the various F_1_ lines. When preparing the 73 F_1_ genotypes included in the mapping experiments, we recorded which batches of seeds came from which pods. Thus, we could later check whether the pollen that fertilized a given pod typically came from a single pollen parent or from multiple parents (Supporting File 1). In the field where the seed was collected in 2013, 38 apios lines were growing in relatively close proximity (~ 10 m × 20 m area), so numerous pollen parents were possible. Controlled manual pollinations have been difficult in apios (Bruneau and Anderson 1988; Blackmon, personal observation), so this way of establishing parentage was not attempted.

Among the most likely pollen parents for the 73 F_1_ lines, 18 parents were identified. Significantly, this list did not include the maternal parent 2155, suggesting that selfing did not occur in the generation of this mapping population. Multiple pollen parents were also frequently observed to fertilize seeds from a single pod, indicating that pollinators were visiting multiple genotypes and depositing pollen from several pollen parents on any given pistil. Of 13 pods with multiple seeds making it into the final mapping population (the average seeds per pod were 3.1 for the 73 mapping genotypes), 11 had multiple pollen parents, 2 had seeds from a single pollen parent, and 4 had two or more seeds from one parent (Supporting File 1). The pod with the most seeds represented in the population (*n* = 6) had two pollen parents: 3 seeds were sired by accession AA-807 and 3 by accession AA-2003. Among the pollen parents, some appeared more frequently: three genotypes (AA-2003, AA-807, and AA-2110) accounted for 53% of the fertilization events (Supporting File 2). Including the next two most frequent paternal genotypes (AA-2065 and AA-1943) accounted for 70% of the fertilization events. We conclude from these patterns that the distributions of pollen parents were non-random, and that seeds in a given pod are more likely to share a pollen parent than to have disparate parents—but that “multiple paternity” of seeds in a pod is, nevertheless, a common pattern.

### Linkage mapping

The high-quality markers from the previous step were converted into a mapping format for the purpose of linkage analysis. Given the large number of distinct pollen parents, the markers with male genotype-specific segregation patterns (‘*nn x np*’) were not used for further analysis. The remaining maternal segregating type (‘*lm x ll*’) and heterozygous type (‘*hk x hk*’) loci were separately analyzed for recombination analysis and detection of linkage.

The initial screening of marker loci indicated some markers showing identical segregation patterns and belonged to the same recombination bins. The most-informative marker with the least missing data was selected from each bin to represent the recombination event on the map. The remaining marker loci were grouped to identify high confidence linkage groups that match apios basic chromosome number (2*n* = 2*x* = 22) and remain stable even at an increased linkage threshold criterion. Unfortunately, the loci with maternal testcross segregation types (‘*lm x ll*’) were unable to form stable linkage groups at different threshold criteria. These marker types either form a very few linkage groups (*n* = 2–4) with large number of markers contained in each of these groups, or they split into large number of small-sized groups (no. of markers ≤ 10), even after a single point increase in the threshold criterion. Thus, the ‘*lm x ll*’ markers were considered unsuitable and loci with only F_2_ segregating types (‘*hk x hk*’) were used to generate the linkage groups. The process of assigning markers into distinct groups was repeated several times to eliminate unlinked markers.

**Fig. 3:**
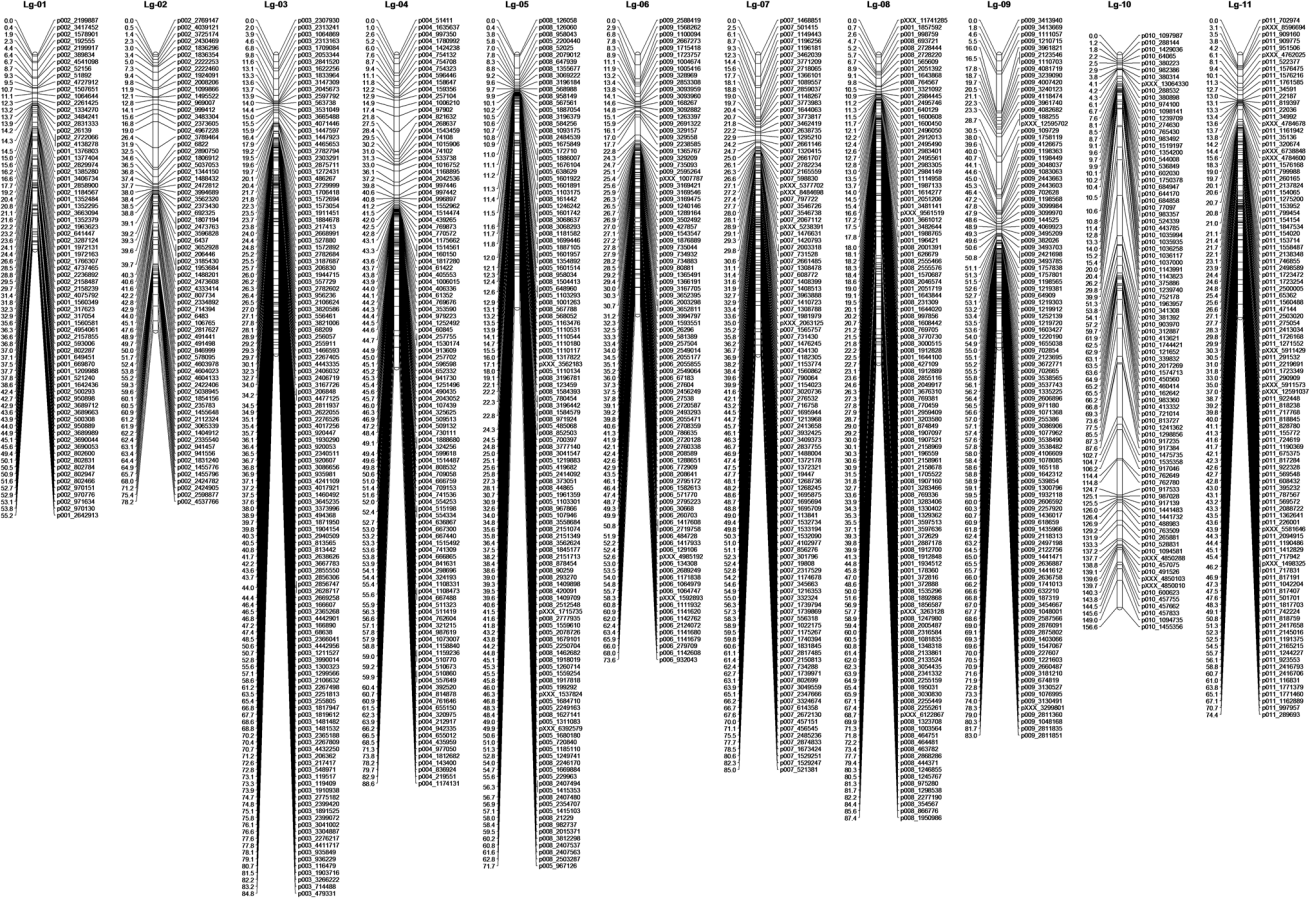
Linkage map of apios (LOD 4) having 1121 recombinationally unique loci distributed on 11 linkage groups

An initial set of 4309 markers was used to calculate recombination frequency and marker order for apios map construction. We were unable to assign positions for 3188 markers that fell under the defined linkage criteria (LOD = 4, maximum recombination frequency = 0.35) and the final apios genetic map consisted of the remaining 1121 marker loci that spanned a total map length of 938.6 cM (Fig. 3, Supporting file 2). The 3188 markers could have been assigned a position using lower threshold criteria, but doing so would have created erroneous marker placements by forcing a map position at a lower threshold value. The size of linkage groups for the more selective marker set (1121 markers) varied from a minimum of 55.2 cM (Lg-01) to a maximum value of 156.6 cM (Lg-10) (Table 2). The average linkage group length was 85.3 cM across the map. The number of loci varied considerably in each linkage group. For instance, Lg-02 and Lg-01 had the fewest markers (72 and 73, respectively), while Lg-03 and Lg-05 had the most (128 and 124, respectively). The average distance between pair-wise marker loci ranged from 0.6 cM for Lg-05 to 1.8 cM for Lg-10. Some of the pair-wise loci also spanned large gaps of varying sizes in different linkage groups. For example, the largest gap in Lg-10 spanned 15.1 cM, while the Lg-03 spanned a gap size of 3.1 cM. Some gaps were flanked by closely spaced markers, leading to an uneven marker distribution across such regions. Many other regions had more uniform marker distributions.

**Table 2:** Marker distribution on linkage groups of apios

Linkage group	Mapped loci	Linkage group size (cM)	Average loci distance (cM)	Maximum gap size (cM)
Lg-01	73	55.2	0.8	3.8
Lg-02	71	78.2	1.1	7.5
Lg-03	128	84.8	0.7	3.1
Lg-04	112	88.6	0.8	5.7
Lg-05	124	71.7	0.6	8.9
Lg-06	93	73.6	0.8	5.6
Lg-07	110	85.0	0.8	4.4
Lg-08	117	87.4	0.8	5.3
Lg-09	104	83.0	0.8	10.5
Lg-10	87	156.6	1.8	15.1
Lg-11	102	74.4	0.7	3.8
Sum	1121	938.6		
Average	101.9	85.3		

The JoinMap linkage map was independently validated by generating a separate map with the aid of MultiPoint software. From the 1121 markers used to produce the JoinMap map, 506 ‘skeleton’ markers were identified and used to generate 11 final linkage groups (Supporting File 3). These linkage groups ranged in length from 159.2 to 342.6 cM; overall map length was 2464.7 cM (Table S4). Compared to the JoinMap-generated map, the MultiPoint-generated linkage groups were substantially similar in locus order. Differences in map length were attributed to the distance modifier implemented in JoinMap software.

### Synteny between apios and related warm-season legume genomes in the Phaseoleae

To understand the patterns of genome evolution and structural conservation among several genomes in the Phaseoleae and test the robustness of the genetic linkage map built in this study, we analyzed genome-wide syntenic relationships between apios and four closely related legume species by homology match and dot-plot visualization. The nucleotide sequences spanning 200 bp around each of the 1121 mapped loci were extracted (Supporting file 4) and matched against genome sequences from *P. vulgaris* (Pv), *G. max* (Gm), *V. radiata* (Vr), and *V. angularis* (Va). Based on the alignment score, top matches were retained from each species except soybean. In soybean, the top two matches were retained, consistent with the relatively recent polyploidy event within the *Glycine* genus that resulted in two subgenomes (Schmutz et al. 2010). The alignment information was used to determine the homologous chromosomes (those showing the largest number of matching sequences), syntenic blocks, and genome co-linearity between the five species.

The number of homologous sequences was highest between apios and Gm (1379 matches) followed by Pv (571 matches 50.9%), Vr (470 matches, 41.9%), and Va (360 matches, 32.1%) at the defined threshold level (Table S5, Supporting file 5). In soybean, 572 marker sequences (41.5%) out of 1379 matches showed a second high-quality correspondence with the soybean sequences, and the remaining 807 marker sequences (58.5%) had unique matches against single soybean sequences. The average number of matched apios loci ranged from ~ 31 (adzuki bean) to ~ 125 (soybean) per linkage group. Marker loci in most of the apios linkage groups matched to a single homologous chromosome in Pv, Vr, and Va, while a minimum of two homologous chromosomes were identified relative to Gm. For example, apios linkage groups Lg-02, Lg-03, Lg-04, Lg-07, Lg-09, and Lg-10 have a direct one-to-one correspondence with single Pv, Vr, and Va chromosomes (Table 3). The proportion of markers that did not follow these regular syntenic patterns with other legume chromosomes was relatively low in these apios linkage groups. In contrast, four apios linkage groups (Lg-01, Lg-05, Lg-06, and Lg-08) showed substantial syntenic rearrangements. The mapped loci on these linkage groups were mostly distributed among two chromosomes in at least one of the four legume species. The mapped loci on Lg-01 matched to two chromosomes in Pv (Pv01, Pv02), Vr (Vr03, Vr07), and Va (Va02, Va04), while having matches to four chromosomes in Gm (Gm05, Gm03, Gm07, and Gm08). Similar results were obtained in the case of Lg-06, with four corresponding linkage group matches with Gm (Gm04, Gm06, Gm09, and Gm15). Interestingly, Lg-05 and Lg-08 displayed a unique distribution of matching loci in the four different legume species. Lg-05 showed corresponding homologous matches to one chromosome in Vr, two chromosomes in Pv and Va, and five chromosomes in Gm. Lg-08 showed a slightly different pattern than Lg-05 and had corresponding single chromosome matches against Vr and Va genomes, two homologous matches in Pv, and four homologous matches in Gm. This analysis clearly indicated that apios had apparent structural conservations to various legume species that is further extended at the level of chromosome integrity similar to specific legume species.

**Table 3:** Proportion of loci within each linkage group in apios having match with corresponding chromosomes in *P. vulgaris*, *G. max*, *V. radiata*, and *V. angularis*

Apios linkage groups	*P. vulgaris* chromosomes	*G. max* chromosomes	*V. radiata* chromosomes	*V. angularis* chromosomes
Lg-01	Pv01 (**8**), Pv02 (**34**)	Gm03 (8), Gm05 (**19**), Gm07 (**20**), Gm08 (**22**), Gm13 (9), Gm19 (9)	Vr03 (**5**), Vr07 (**21**)	Va02 (**7**), Va04 (**4**)
Lg-02	Pv02 (**35**)	Gm01 (**47**), Gm02 (**20**), Gm09 (8)	Vr11 (**18**), Vr07 (3)	Va04 (1), Va06 (2), Va07 (2), Va08 (2), Va11 (1)
Lg-03	Pv03 (**71**)	Gm02 (9), Gm05 (**23**), Gm07 (**10**), Gm13 (**35**), Gm17 (**84**)	Vr07 (**65**)	Va11 (**70**)
Lg-04	Pv04 (**59**)	Gm01 (8), Gm02 (4), Gm05 (**19**), Gm07 (**17**), Gm09 (**36**), Gm13 (**17**), Gm16 (**24**), Gm19 (**31**)	Vr01 (**50**), Vr07 (4)	Va04 (**40**)
Lg-05	Pv05 (**29**), Pv08 (**29**)	Gm02 (3), Gm07 (4), Gm08 (9), Gm09 (**18**), Gm12 (**16**), Gm13 (**21**), Gm15 (**20**), Gm18 (**29**)	Vr04 (**23**), Vr05 (**11**)	Va04 (**18**)
Lg-06	Pv06 (**8)**, Pv09 (**33**)	Gm04 (**26**), Gm06 (**39**), Gm09 (**15**), Gm13 (5), Gm15 (**20**)	Vr05 (**12**), Vr10 (**11**)	Va03 (**9**), Va09 (**10**)
Lg-07	Pv07 (**55**)	Gm02 (**10**), Gm10 (**73**), Gm13 (4), Gm15 (3), Gm20 (**50**)	Vr08 (**48**)	Va06 (**55**)
Lg-08	Pv01 (**34**), Pv08 (**30**), Pv03 (4)	Gm02 (**38**), Gm06 (3), Gm09 (4), Gm13 (**10**), Gm14 (**74**), Gm17 (**18**)	Vr06 (**58**)	Va01 (**58**)
Lg-09	Pv09 (**47**)	Gm04 (**57**), Gm06 (**60**)	Vr05 (**48**)	Va05 (**36**)
Lg-10	Pv10 (**36**), Pv08 (3), Pv06 (3)	Gm01 (**16**), Gm03 (**28**), Gm07 (**36**), Gm08 (7), Gm16 (**10**), Gm18 (3)	Vr09 (**27**)	Va08 (**15**)
Lg-11	Pv11 (**45**)	Gm06 (**18**), Gm09 (**13**), Gm11 (**18**), Gm12 (**54**), Gm13 (4)	Vr02 (**39**)	Va02 (**6**), Va05 (**7**)

The apios marker sequences were matched against the genome sequences of four legume species using blastn (e-value < 1e−10). The number in the brackets represents total apios matches to a particular chromosome in other species. The bracketed letters in bold represent the maximum hits. The entries with less than three hits per chromosome are not presented in this table except Lg-02 matches against *V. angularis*

**Fig. 4:**
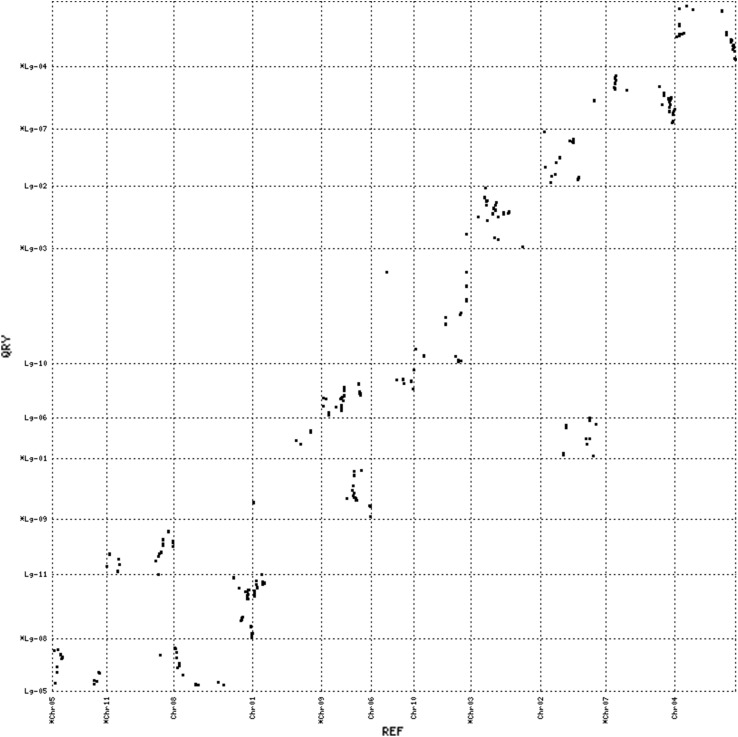
Dot plot of synteny comparisons between apios and *Phaseolus vulgaris*

**Fig. 5:**
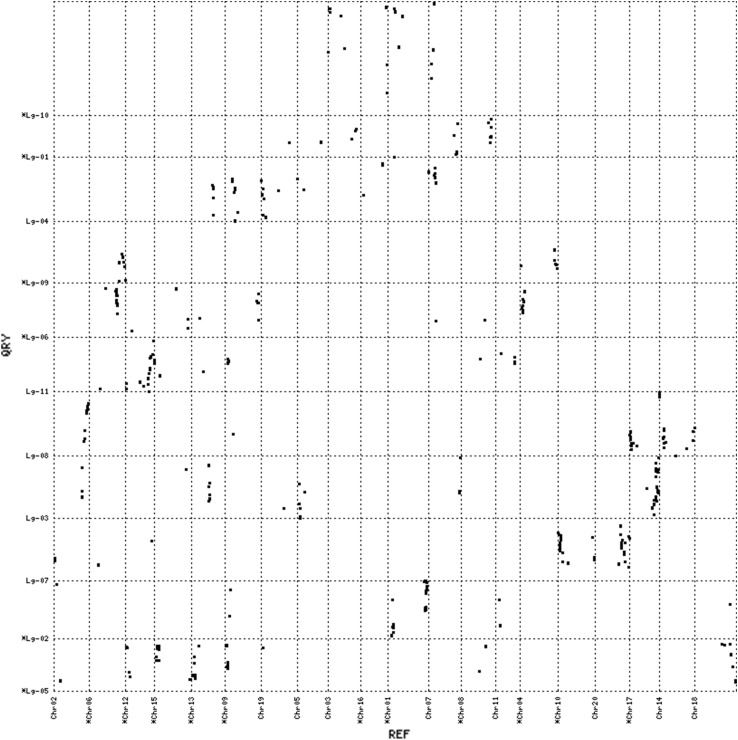
Dot plot of synteny comparisons between apios and *Glycine max*

**Fig. 6:**
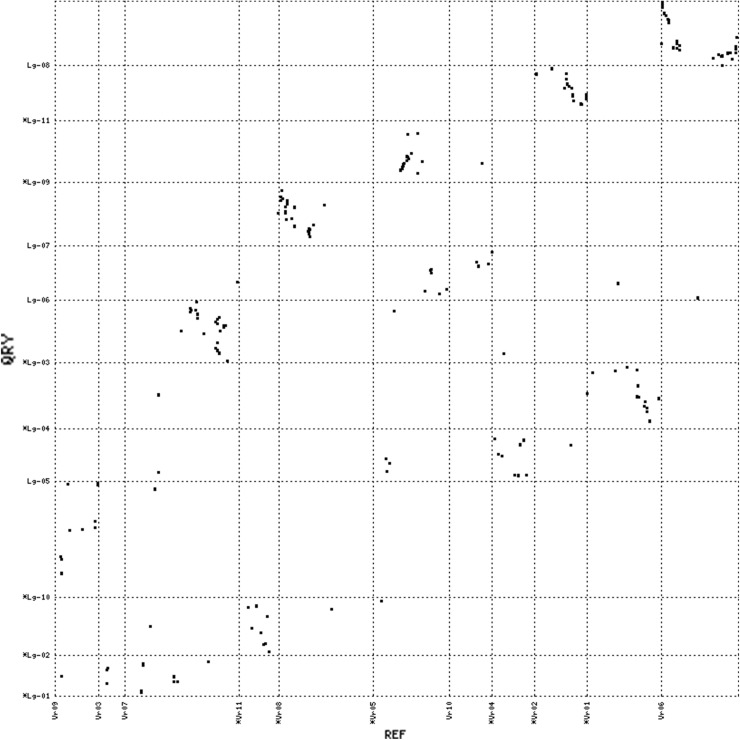
Dot plot of synteny comparisons between apios and *Vigna radiata*

**Fig. 7:**
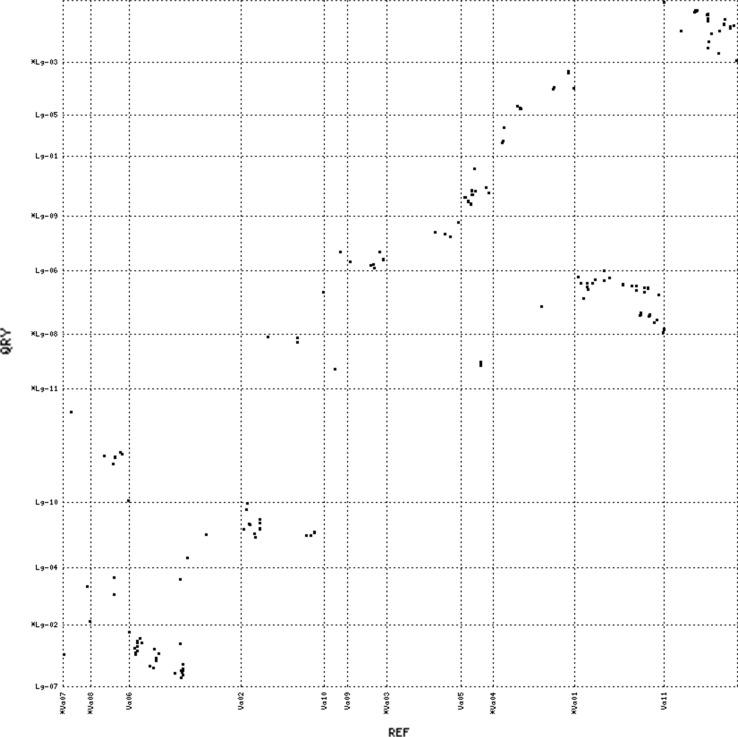
Dot plot of synteny comparisons between apios and *Vigna angularis*

The syntenic relationships between apios and the four legume species were further examined at the level of gene order conservation. We defined the conserved gene order as having at least three mapped loci in apios that showed collinearity with the physical coordinates of the matched chromosome in a contrasting legume species. Apios exhibited distinct collinear blocks of varying sizes against all four of the comparison legumes (Figs. 4, 5, 6, 7 and Supporting file 5). The total collinear blocks of synteny shared were highest against Gm (113) followed by Pv (44), Vr (42) and Va (35), with an average shared block size ranging from ~ 2.97 cM against Gm to ~ 4.88 cM against Vr. Almost every apios linkage group shared collinear regions with at least one of the legume species. However, the extent and pattern of collinearity was different across chromosomes. There were fewer large collinear blocks (≥ 5 loci in the same block) between apios and Va (3), while the number of such blocks was highest against Gm (15). Among all the apios linkage groups, only Lg-03 shared large collinear blocks with each of the four species, while Lg-04 exhibited collinearity with three species (Pv, Gm, and Va). The largest collinear block was detected on apios Lg-10 that covered about 47.5 cM genetic distance against Gm. However, the largest number of closely segregating loci (9) within a syntenic block was observed against comparison with Pv, covering about 11 Mbs sequence on Pv chromosome 3. In all cross comparisons, apparent inversions were observed within the collinear blocks.

There are several collinear regions spanning more than 15 Mbs physical distance against the four legume species (Supporting file 5). For instance, the largest collinear block relative to Pv covered ~ 39 Mbs (Lg-04), followed by a block covering ~ 38 Mbs (Lg-11). Similarly, the largest collinear blocks against mung bean and adzuki bean were ~ 31 Mbs and ~ 27 Mbs, respectively. The blocks covering large physical distances in these legumes were mostly spanned by 3–4 collinear loci, which suggests that the respective markers were most likely located in regions of low recombination. Discounting such large gaps (> 15 Mbs) to avoid bias in the calculation of physical coverage for Pv, Gm, and Vr, the remaining collinear blocks covered an average of ~ 1.1 Mbs (Gm) to ~ 3.6 Mbs (Va) of physical distance, while ratio of physical distance to the genetic distance (bp/cM) ranged from 0.51 in Gm to 1.3 in adzuki bean (Table S6).

## Discussion

This study provides the initial characterization of the chromosomal linkage patterns in the tuberous legume, *Apios americana* (apios). High-throughput sequencing can provide a significant advantage to building genomic resources in less-characterized species (Wang et al. 2009; Schliesky et al. 2012; Belamkar et al. 2016). We used an RNA-seq-based approach to generate a high-quality reference transcriptome for identifying informative SNP markers in our mapping population. The large number of markers available in high-throughput sequencing enabled us to overcome several challenges in this species and in our mapping population: first, that the species appears to be obligately or at least substantially outcrossing, and mapping approaches must take this into account; and second, that we began with a maternal line crossed with multiple pollen parents to generate our mapping population. This means that the segregation pattern in the male parents, though present, would be complex due to genetic variation among them. We were able to evaluate many potential markers unambiguously matching the maternal genotype, and to use a one-way pseudo-testcross strategy to develop a linkage map in apios. We were also able to infer probable pollen parents for each of the F_1_ population line, by genotypic comparison with all potential pollen donors.

The apios reference transcriptome was de novo assembled and refined through various filtering steps to remove low-quality and misassembled transcripts. The apios transcriptome showed a high level of coding sequence matches against the related warm-season legumes that defines the robustness and completeness of this transcriptome. These results were consistent with de novo assembled transcriptomes in other plant species (Tao et al. 2012; Liu et al. 2013; Ranjan et al. 2014). We used the refined apios assembly for the identification of SNPs, because this assembly produced a larger proportion of high-quality SNPs than the original unrefined assembly. An elimination of repetitive and misassembled sequences might cause the increased number of high-quality SNPs, probably due to increased precision of read alignment and variant detection. Although we identified a large number of SNPs in this population, the proportion of informative SNPs was higher in the refined set. A large number of SNPs remained unused due to segregation patterns that were specific to the pollen source or the lack of useful recombination among them. A more advanced population with more individuals would enable use of more SNPs. Despite these challenges, we generated a linkage map consisting of 1121 unique loci spread across 11 linkage groups. Few regions on the apios map contain large gaps. Some of these gaps may represent gene-poor regions or regions of low polymorphism. The cause(s) of these gaps may become clearer when a reference genome sequence becomes available for this species.

Although the legumes are a large and diverse family of flowering plants, extensive genomic sequence conservation exists among related species (McConnell et al. 2010; Cannon et al. 2006; Gepts et al. 2005; Zhu et al. 2005). Our analysis supports this observation as inferred by numerous extended syntenic matches between apios and related crop legumes. The number of homologous sequences in apios was comparatively higher against *P. vulgaris* and *G. max* than the two *Vigna* species, although genome architecture, assembly size, and quality should be taken into account while making such interpretations. For instance, many evolutionary events were accompanied by genome duplications and gene losses (Bowers et al. 2003; Vision et al. 2000; Krylov et al. 2003). These events lead to uneven sequence content and likewise affect the detection of homologous matches between the two species compared during synteny analysis. Whole-genome duplication in soybean (Schmutz et al. 2010; Cannon et al. 2006; Shoemaker et al. 1996) provides, in many instances, two homologous matches for apios sequences. Similarly, the quality and completeness of sequenced genomes can influence the comparison between two species. Some of the recently sequenced genomes still lack a considerable amount of sequencing information. For example, the draft genome of *V. angularis* is lacking ~ 25% of the estimated total genome (Kang et al. 2015). Such information should be accounted for during comparison of syntenic relationships against more than one species.

Map-to-sequence comparisons identified a clear correspondence between many apios linkage groups and the chromosomes in other legume genomes. Nine out of eleven apios linkage groups exhibited such clear one-to-one match with the comparison species, suggesting a high level of chromosome conservation among these warm-season legume species. Small-scale translocations, missing sequence information (incomplete genome sequences), or small duplication events with a lost ancestral sequence most likely explain the relatively few matches to additional chromosomes in these nine linkage groups. In contrast, two apios linkage groups showed corresponding matches in marker loci to two or more chromosomes in at least one of the other legumes. Lg-01 showed correspondence with two chromosomes in Pv and both *Vigna* species, and had four matching chromosomes against Gm. Similarly, Lg-06 showed clear matches against two chromosomes in Pv, Vr, and Va, but had four corresponding chromosomal matches with Gm. Lg-05 and Lg-08 also showed correspondence to more than two chromosomes in soybean. The genome duplication history can explain this observation in soybean (Schmutz et al. 2010). It further suggests that complex translocation events may have occurred during the evolution of apios Lg-01, Lg-05, and Lg-06 in comparison to the other warm-season legumes. Similarly, Lg-08 represents an interesting scenario of chromosome evolution in warm-season legumes. It contained marker loci with significant matches to single chromosomes in *Vigna* species, two chromosomes in Pv, and four chromosomes in Gm, which raises the possibility of a translocation event happening after the divergence of apios, Vr, and Va from Pv and Gm. This translocated segment would have undergone duplication in soybean later on, which leads to matching of Lg-08 to the four chromosomes in Gm. A closer analysis of Lg-08 syntenic regions in the genomes of the four legumes using the synteny viewer provided by the Legume Information System (Dash et al. 2015) confirms this observation. A translocation event is apparent between the Pv and Gm against the two *Vigna* species. This observation suggests that apios Lg-08 evolved similar to the *Vigna* species. These genomic rearrangements can be confirmed either with high-quality genome sequencing with additional genetic mapping, or comprehensive karyotype analysis with homologous markers and fluorescent in situ hybridization (FISH) experiments. Nevertheless, use of apios in synteny analysis with other species in the Phaseoleae is informative in that apios diverged prior to the radiation of the other comparison species, so that it may serve as an outgroup and a potential indicator of progenitor states of gene order. This approach helped suggest possible translocations on Lg-01, Lg-05, Lg-06, and Lg-08, which most likely occurred after its divergence from other related legumes.

The genetic map constructed in this study provides tools for additional steps to be taken towards the domestication of apios. Many gene-based polymorphic markers were obtained that could be utilized for marker-assisted selection, linking genes to phenotype, and finally map-based cloning of identified genes. Moreover, the high average marker density obtained here should provide the basis of relatively narrow QTL intervals for several economically important traits such as growth habit, mother tuber size, child tuber size, flowering time, etc., for which selection and improvement are desirable. The previous information from well-studied legumes in the Phaseoleae can facilitate the transfer of genetic information by identifying apios homologs and their locations on the current genetic map. It will help target the candidate genes responsible for the phenotypic variation of many suitable breeding traits.

The mapping results were also useful in clarifying several features of reproductive biology in apios. We observed no instances of selfing in apios among all progeny. Bruneau and Anderson (1988) reported low rates of seed set in self-pollinated plants with no seeds in 92% of tripped flowers and with few seeds per pod (average of 1.9 for selfed pods vs. 6.3 outcrossed pods). Regarding the apparent capacity for self-pollination in some but not all apios genotypes, they conclude “although *A. americana* seems self-compatible, fruit and seed set from self-pollination is low, and not all individuals that produce fruit from cross-pollination yield fruit from selfing” (Bruneau and Anderson 1988). Because no instances of selfing were observed among the 72 F1 seeds and plants from the 2155 maternal line in our mapping cross, this line may be particularly recalcitrant to self-fertilization. Interestingly, we did observe multiple pollen parents for seeds within a single pod—presumably, due to pollinators visiting flowers from several plants and depositing the mixed pollen on the pistils of subsequent flowers. Although 18 pollen parents were deduced, seven pollen parents were highly over-represented in the progeny (accounting for 70% of the fertilizations). This might have been due to flower abundance or developmental stage, proximity, or pollen viability or compatibility.

The apios linkage map can be used to analyze diversity in different wild and improved apios lines. Such analyses can allow the use of polymorphic sites and linkage information obtained in a single cross to multiple genotypes to facilitate the association of polymorphic markers with traits of interest. The ordered marker information can be utilized to target regions of agronomic importance in a diverse genotype panel for the identification of the candidate genes. Although the genomic efforts in apios have been minimal to date, this map could serve for ordering genome scaffolds and their refinement.

## Conclusion

We characterized the genome-wide synteny patterns in phaseoloid legumes by constructing a transcript-based linkage map for *Apios americana*, which is an early diverging member of the Phaseoleae, and of interest, because it is a potential food crop. An experimental approach was described that developed a reliable reference transcriptome assembly, SNP calling, and genotyping for this species. The large number of markers obtained in this study provided a preliminary glance at recombination in apios and its syntenic relationships with common bean, soybean, mung bean, and adzuki bean. A translocation event between apios, mung bean, and adzuki bean was confirmed against the common bean and soybean genomes. This study has not only generated a large number of molecular markers for genetic characterization of several economically important traits in apios, but also provided helpful information to clarify the history of genome structure of species in the Phaseoleae.

### **Author contribution statement**

JS conducted the mapping analysis and drafted the manuscript. SRK managed field plots and germplasm. VB collected tissue, isolated RNA, and conducted parentage analysis. TM conducted cytological and microscopy experiments. MNN conducted mapping analysis using MultiPoint software. ADF did variant calling relative to the reference transcriptome assembly. WJB managed the crosses and selections resulting in the lines used in this study, and grew and collected the seed used in the mapping experiment. SBC, VB, and JS conceived and guided the experiments. All authors reviewed and approved the manuscript.

## Electronic supplementary material

Below is the link to the electronic supplementary material.

Supplementary material 1 (TIFF 758 kb) **Supplementary Fig.** **1**. A chromosomal spread from a root preparation from apios, cv. AA-2155

Supplementary material 2 (XLSX 17 kb) **Supplementary File 1**. Analysis of parentage based on genotyping of progeny, maternal parent, and potential paternal parents

Supplementary material 3 (XLSX 339 kb) **Supplementary File 2**. Linkage map developed using Joinmap software with genotype scores

Supplementary material 4 (XLSX 157 kb) **Supplementary File 3**. Linkage map developed using MultiPoint software comprising 506 skeleton markers and the graphical genotypes in the mapping population (n = 73)

Supplementary material 5 (XLSX 121 kb) **Supplementary File 4**. Sequences around the SNP markers mapped to generate apios linkage map with Joinmap

Supplementary material 6 (XLSX 384 kb) **Supplementary File 5**. Supporting homology information for genome synteny comparisons

Supplementary material 7 (DOCX 24 kb)
