# Supplementary figures and images for: A transcriptome-SNP-derived linkage map of Apios americana (potato bean) provides insights about genome re-organization and synteny conservation in the phaseoloid legumes
Source: Theor Appl Genet. 2017 Oct 25;131(2):333–51. doi: 10.1007/s00122-017-3004-3 (PMC5787225; doi:10.1007/s00122-017-3004-3)

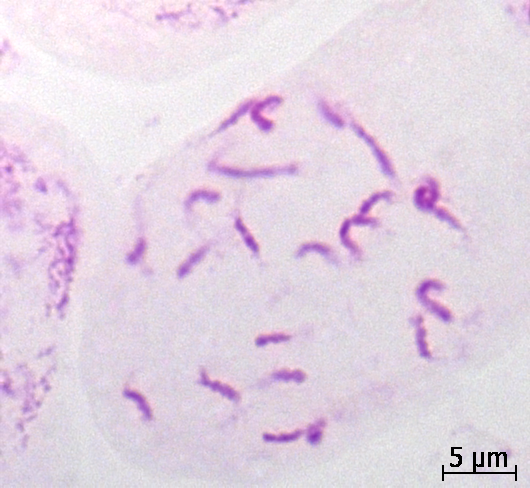

Supplement: Supplementary file 1 — Supplementary material 1 (TIFF 758 kb) Supplementary Fig. 1. A chromosomal spread from a root preparation from apios, cv. AA-2155 [file 122_2017_3004_MOESM1_ESM.tif]
