# Supplementary material for: A transcriptome-SNP-derived linkage map of Apios americana (potato bean) provides insights about genome re-organization and synteny conservation in the phaseoloid legumes
Source: Theor Appl Genet. 2017 Oct 25;131(2):333–51. doi: 10.1007/s00122-017-3004-3 (PMC5787225; doi:10.1007/s00122-017-3004-3)
Supplement: Supplementary file 7 — Supplementary material 7 (DOCX 24 kb) [file 122_2017_3004_MOESM7_ESM.docx]

**Table S1**. Comparative analysis of Apios transcriptome assembly (“AA-2155”, refined) with *Glycine max*, *Phaseolus vulgaris*, *Vigna radiata*, *Vigna angularis*

|  | **Total coding Sequences** | **Blast Hits Apios Transcripts** | **Unique Blast Hits** | **No. of Transcripts ( >50% alignment)** | **No. of Transcripts ( >80% alignment)** |
| --- | --- | --- | --- | --- | --- |
| *Glycine max* | 31,638 | 25,740 | 21,535 | 5,218 | 10,927 |
| *Phaseolus vulgaris* | 88,647 | 61,659 | 49,274 | 9,422 | 21,265 |
| *Vigna radiata* | 22,368 | 24,099 | 17,840 | 5,322 | 9,534 |
| *Vigna angularis* | 36,692 | 25,054 | 19,060 | 5,935 | 10,301 |

**Table S2**. Number of unique reads aligned to the original and refined transcript assembly from genotypes “AA-2127” and “AA-2155”. The genotype “AA-2127” was used as a control.

| **Genotype** | **AA-2127 Refined** | **AA-2127 Original** | **AA-2155 Refined** | **AA-2155 Original** |
| --- | --- | --- | --- | --- |
| 2003 | 19,118,841 | 15,395,356 | 20,713,456 | 18,311,015 |
| 2003_RCS | 10,044,384 | 9,291,965 | 10,833,064 | 10,738,697 |
| 52 | 17,777,298 | 17,740,209 | 19,200,418 | 19,184,408 |
| 807 | 19,327,888 | 17,353,716 | 20,738,411 | 19,691,559 |
| 807_RCS | 7,860,720 | 7,342,014 | 8,439,893 | 8,400,388 |

**Table S3**. Single nucleotide polymorphisms (SNPs) detected using the original and refined transcript assembly from genotypes “AA-2127” and “AA-2155”. The genotype “AA-2127” was used as a control.

|  | **Total Number of Variant Calls** | **Number of Variant Calls after Filtering** |
| --- | --- | --- |
| Reduced AA-2127 | 237,325 | 183,363 |
| Unreduced AA-2127 | 497,501 | 110,778 |
| Reduced AA-2155 | 289,320 | 212,769 |
| Unreduced AA-2155 | 504,692 | 136,098 |

**Table S4**. Marker distribution on linkage groups of apios calculated using MultiPoint software.

| **Linkage Group** | **Skeleton loci number ^1^** | **Linkage group size (cM)** | **Average interval size (cM)** | **Maximum interval size (cM)** |
| --- | --- | --- | --- | --- |
| Lg-01 | 35 | 159.2 | 4.5 | 12.4 |
| Lg-02 | 36 | 174.4 | 4.8 | 19.5 |
| Lg-03 | 64 | 305.9 | 4.8 | 16.9 |
| Lg-04 | 50 | 211.7 | 4.2 | 13.8 |
| Lg-05 | 56 | 206.3 | 3.7 | 17.6 |
| Lg-06 | 36 | 176.9 | 4.9 | 26.8 |
| Lg-07 | 57 | 342.6 | 6.0 | 23.6 |
| Lg-08 | 51 | 251.4 | 4.9 | 31.4 |
| Lg-09 | 39 | 204.1 | 5.2 | 20.2 |
| Lg-10 | 39 | 250.7 | 6.4 | 24.0 |
| Lg-11 | 43 | 181.5 | 4.2 | 14.7 |
| **Sum** | **506** | **2464.7** |  |  |
| **Average** | **46** | **224.1** |  |  |

^1^MultiPoint software identifies the highest-quality markers, termed 'Skeleton' markers, which are used to construct the linkage groups.

Redundant and lower quality markers are not used in mapping and are not presented here.

**Table S5**. The number of matches against common bean (*P. vulgaris*), soybean (*G. max*), mungbean (*V. radiata*), and adzuki bean (*V. angularis*) for apios marker loci sequences within each linkage group.

| **Apios Linkage Groups** | **P. vulgaris Matches** | | **G. max Matches** | **V. radiata Matches** | **V. angularis Matches** |
| --- | --- | --- | --- | --- | --- |
| Lg-01 | 42 | 89 | | 29 | 15 |
| Lg-02 | 35 | 87 | | 24 | 8 |
| Lg-03 | 71 | 170 | | 67 | 72 |
| Lg-04 | 60 | 158 | | 55 | 40 |
| Lg-05 | 59 | 125 | | 38 | 22 |
| Lg-06 | 42 | 111 | | 26 | 20 |
| Lg-07 | 59 | 147 | | 52 | 56 |
| Lg-08 | 69 | 152 | | 58 | 59 |
| Lg-09 | 47 | 124 | | 49 | 37 |
| Lg-10 | 42 | 103 | | 31 | 17 |
| Lg-11 | 45 | 113 | | 41 | 14 |
| **Total** | 571 | 1379 | | 470 | 360 |
| **Average** | 51.9 | 125.4 | | 42.7 | 32.7 |
| **Min** | 35 | 87 | | 24 | 8 |
| **Max** | 71 | 170 | | 67 | 72 |
|  |  |  | |  |  |
|  |  |  | |  |  |

**Table S6**. The proportion of physical distance in four legume species over genetic distance in apios.

| **Species** |  | **No. of Collinear Blocks** | **No. of loci per Collinear Block** | **Physical distance (bps) Coverage** | **Genetic distance (cM) Coverage** |
| --- | --- | --- | --- | --- | --- |
| **P. vulgaris** | Maximum | 8 | 9 | 11791952 | 31.44 |
|  | Minimum | 1 | 3 | 32152 | 0.27 |
|  | Average | 4 | 2.65 | 2455114 | 3.14 |
|  | **Bps / cM** |  | **780912** | | |
| **G. max** | Maximum | 20 | 6 | 12715269.5 | 47.57 |
|  | Minimum | 3 | 3 | 163907.5 | 0.34 |
|  | Average | 10 | 1.12 | 1113048 | 2.16 |
|  | **Bps / cM** |  | **514556** | | |
| **V. radiata** | Maximum | 8 | 6 | 11361130 | 37.99 |
|  | Minimum | 1 | 3 | 59167 | 0.31 |
|  | Average | 4 | 2.74 | 3276868 | 4.51 |
|  | **Bps / cM** |  | **726621** | | |
| **V. angularis** | Maximum | 8 | 5 | 14409467.5 | 11.8 |
|  | Minimum | 1 | 3 | 4295 | 0.36 |
|  | Average | 3 | 2.80 | 3582474 | 2.73 |
|  | **Bps / cM** |  | **1314669** | | |
